# Supplementary material for: PINK1 is a target of T cell responses in Parkinson’s disease
Source: bioRxiv. 2024 Feb 12:2024.02.09.579465. Preprint. [Version 1] doi: 10.1101/2024.02.09.579465 (PMC10888789; doi:10.1101/2024.02.09.579465)
Supplement: 1 [file NIHPP2024.02.09.579465V1-supplement-1.pdf]

bioRxiv preprint doi: https://doi.org/10.1101/2024.02.09.579465; this version posted February 12, 2024. The copyright holder for this preprint (which was not certified by peer review) is the author/funder, who has granted bioRxiv a license to display the preprint in perpetuity. It is made available under aCC-BY 4.0 International license.

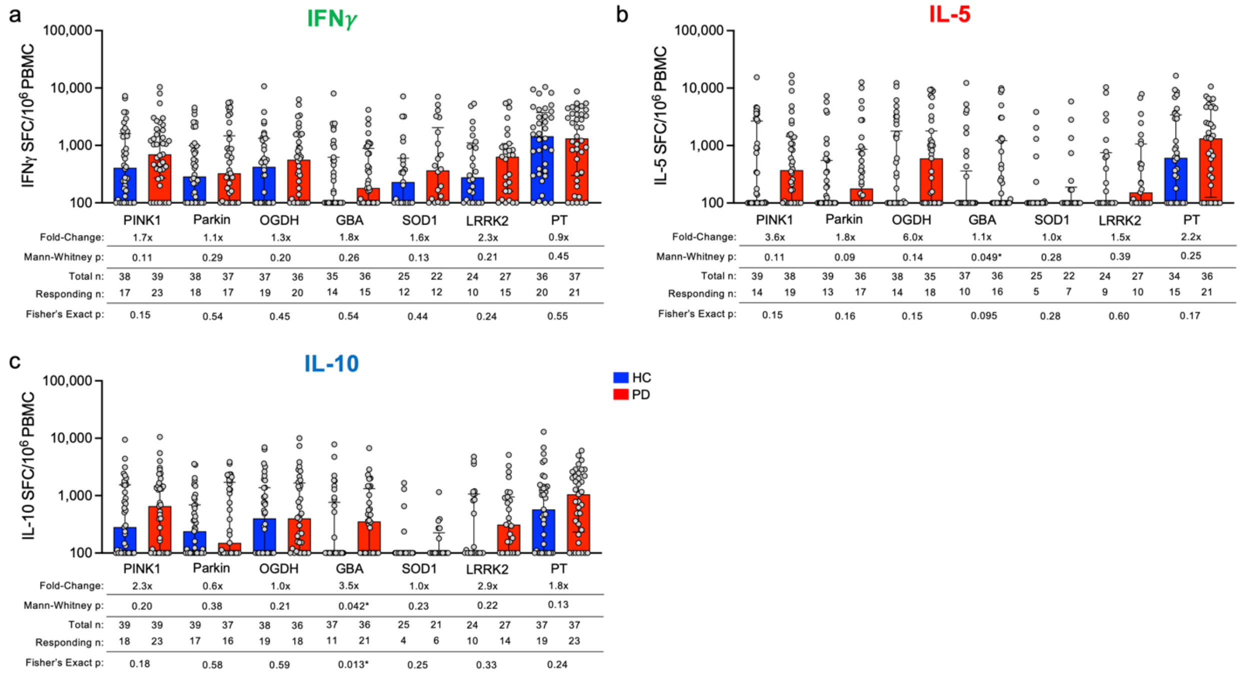

Supplemental Figure 1: Individual cytokine responses towards neuroantigens among PD patients. The magnitude of the individual cytokine response for a) IFNγ, b) IL-5, c) IL-10 by PBMCs from PD and age-matched HC. HC (blue bars) and PD (red bars), each circle representing an individual participant. Median ± interquartile range displayed. Fold-change is in comparison to HC response. One-tailed Mann-Whitney tests were performed between HC and PD antigen-cytokine values. One-tailed Fisher tests were performed using the geometric mean of the HC group for each individual antigen as a cutoff for the test.

bioRxiv preprint doi: https://doi.org/10.1101/2024.02.09.579465; this version posted February 12, 2024. The copyright holder for this preprint (which was not certified by peer review) is the author/funder, who has granted bioRxiv a license to display the preprint in perpetuity. It is made available under aCC-BY 4.0 International license.

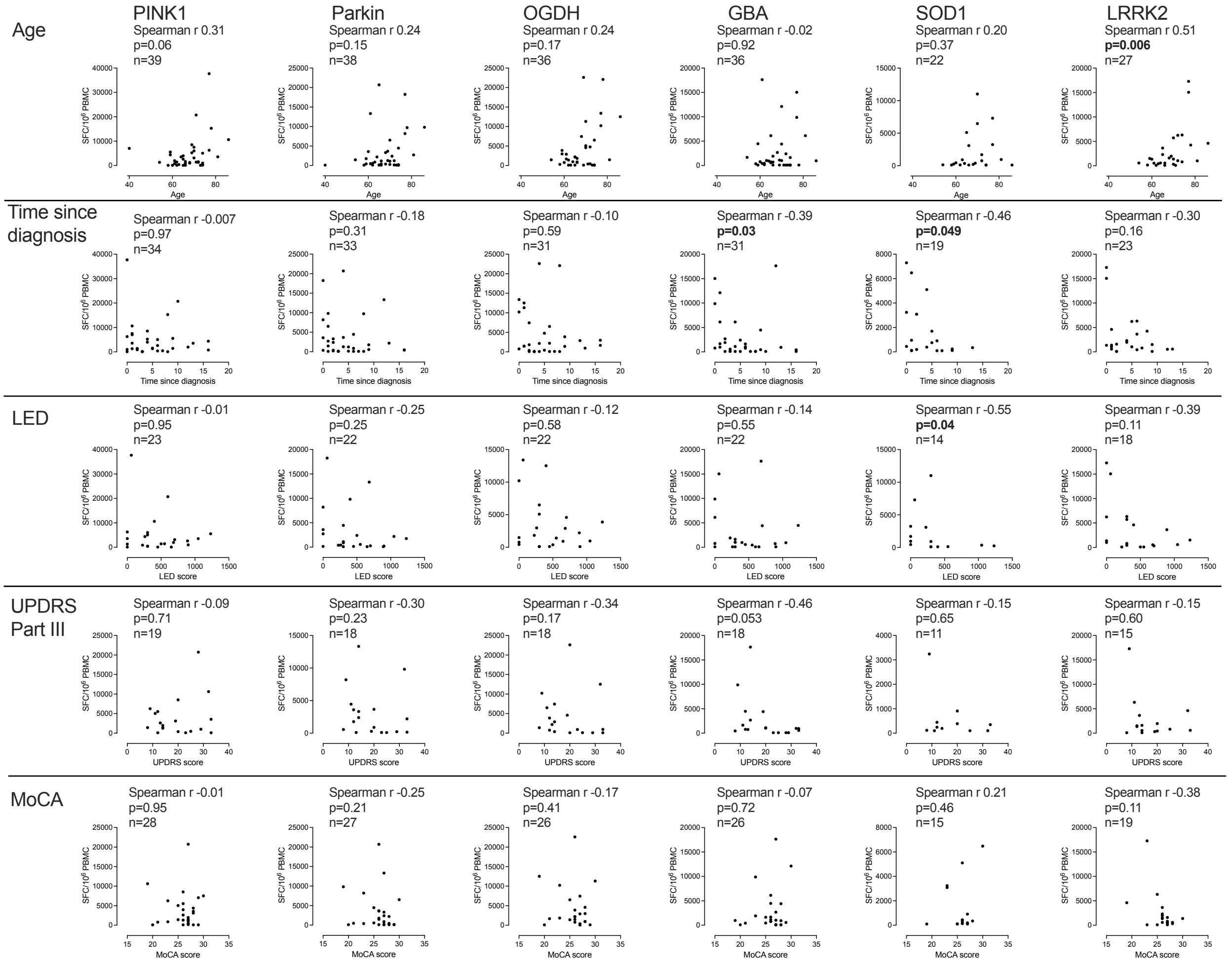

Supplemental Figure 2. Correlation between neuroantigen-specific T cell reactivity and clinical variables. Correlation between age, time from diagnosis, LED, UPDRS part III, and T cell reactivity against MoCA and PINK1, Parkin, OGDH, GBA, SOD1, and LRRK2. T cell reactivity is the sum of the total cytokine response (IFNγ, IL-5, and IL-10) against the respective peptide pools as SFC per 10^6 cultured PBMC. Correlation is indicated by Spearman r and associated p value. Each graph indicates the number of PD patients with the specific clinical variable and T cell reactivity measurement.

bioRxiv preprint doi: https://doi.org/10.1101/2024.02.09.579465; this version posted February 12, 2024. The copyright holder for this preprint (which was not certified by peer review) is the author/funder, who has granted bioRxiv a license to display the preprint in perpetuity. It is made available under aCC-BY 4.0 International license.

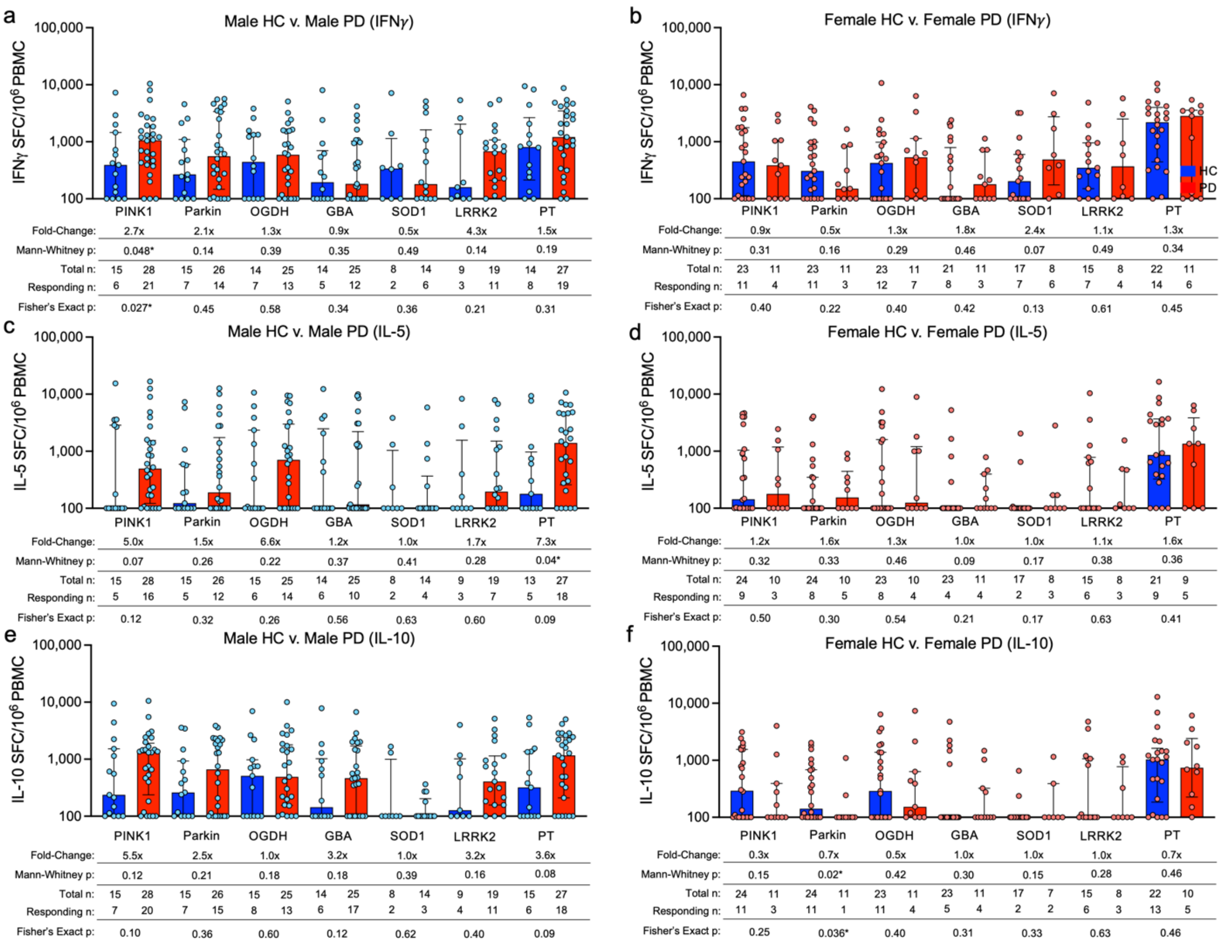

Supplemental Figure 3. Individual cytokine responses among male and female PD patients. Magnitude of the individual cytokine response for a,b) IFNγ c,d) IL-5 e,f) IL-10 among PD and age-matched HC male and female PBMCs. HC (blue bars) and PD (red bars), each circle representing an individual participant. Median ± interquartile range displayed. Fold-change is in comparison to HC response. One-tailed Mann-Whitney tests were performed between HC and PD antigen-cytokine values. One-tailed Fisher tests were performed using the geometric mean of the HC group for each individual antigen as a cutoff for the test.

bioRxiv preprint doi: https://doi.org/10.1101/2024.02.09.579465; this version posted February 12, 2024. The copyright holder for this preprint (which was not certified by peer review) is the author/funder, who has granted bioRxiv a license to display the preprint in perpetuity. It is made available under aCC-BY 4.0 International license.

Supplemental Table 1: Cohort characteristics

	HC<br>n = 39	PD<br>n = 39	*p* value[b]
**Sex (male/female)**			
Male	15	28	
Female	24	11	0.003
**Age in years (mean, SD):**			
Total	66 ± 7	67 ± 8	0.57
Male	68 ± 6	67 ± 9	0.56
Female	64 ± 7	68 ± 5	0.22
**Caucasian (%):**	87 (34/39)	95 (37/39)	
**Time (years) since PD Diagnosis (mean, range; n):**			
Total	N/A	5 (0-16); 34	
Male	N/A	4.9 (0-16); 26	
Female	N/A	5.2 (2-9); 8	
**MDS-UPDRS Part III[a] (mean, SD; n):**			
Total	N/A	19.4 ± 8.5; 19	
Male	N/A	19.9 ± 8.8; 15	
Female	N/A	17.8 ± 6.7; 4	
**MoCA Score[a] (mean, SD; n):**			
Total	N/A	26.0 ± 2.6; 28	
Male	N/A	26.3 ± 2.5; 21	
Female	N/A	25.1 ± 2.8; 7	
**LED (mean, SD; n):**			
Total	N/A	450 ± 350; 23	
Male	N/A	490 ± 371; 17	
Female	N/A	338 ± 233; 6	

[a]UPDRS (III) & MoCA collected at CUMC and UCSD.
[b]Two-tailed Chi-square test comparing the number of male vs. female participants in each cohort, two-tailed Mann-Whitney test comparing age between cohorts
MDS-UPDRS; Movement Disorder Society- Unified Parkinson's Disease Rating Scale
MoCA; Montreal Cognitive Assessment
LED; Levodopa equivalent dose
